# First instance of settlement by cryopreserved coral larvae in symbiotic association with dinoflagellates

**DOI:** 10.1038/s41598-019-55374-6

**Published:** 2019-12-11

**Authors:** Luca Cirino, Zhi-Hong Wen, Kevin Hsieh, Cheng-Liang Huang, Qi Lun Leong, Li-Hsueh Wang, Chii-Shiarng Chen, Jonathan Daly, Sujune Tsai, Chiahsin Lin

**Affiliations:** 1 Department of Marine Biotechnology and Resources, National Sun Yai-sen University, Kaohsiung, Taiwan; 2 He Wei Precision Company Limited, Hsinchu, Taiwan; 3 Department of Applied Chemistry, National Chiayi University, Chiayi, Taiwan; 4 Smithsonian Conservation Biology Institute, Washington, USA; 5 Hawaii Institute of Marine Biology, Hawaii, USA; 6 Department of Post Modern Agriculture, Mingdao University, Chang Hua, Taiwan; 7 National Museum of Marine Biology & Aquarium, Pingtung, Taiwan; 8 Institute of Marine Biology, National Dong Hwa University, Pingtung, Taiwan

**Keywords:** Reproductive biology, Cell biology

## Abstract

Coral reefs are suffering on a global scale due to human impacts, thereby necessitating cryopreservation efforts. The objective of this study was to develop a suitable vitrification and laser warming protocol for larvae of the scleractinian coral *Seriatopora caliendrum*, which inherit their dinoflagellate algal symbionts vertically. Toxicity experiments were conducted with the cryoprotectants (CPAs) ethylene glycol (EG), propylene glycol (PG), dimethyl sulfoxide (DMSO), glycerol (GLY), and methanol (METH; listed in order from least to most toxic), and larvae were subjected to vitrification and laser warming using 2 M EG + 1 M PG and 2 M EG + 1 M DMSO. Vitrification and laser warming (300 V, 10 ms pulse width, 2 mm beam diameter) using a vitrification solution of 2 M EG + 1 M PG, 40% w/v Ficoll, and 10% v/v gold nanobars (GNB) at a final concentration of 1.2 × 10^18^ GNB/mL and a characteristic wavelength of 535 nm resulted in larvae with vitality and settlement percentages of 55 and 9%, respectively. This represents the first successful instance of cryopreservation of coral larvae that proceeded to settle upon warming, and suggests that the vitrification and ultra-fast laser warming approach may be applicable to other threatened marine species.

## Introduction

**Figure 1:**
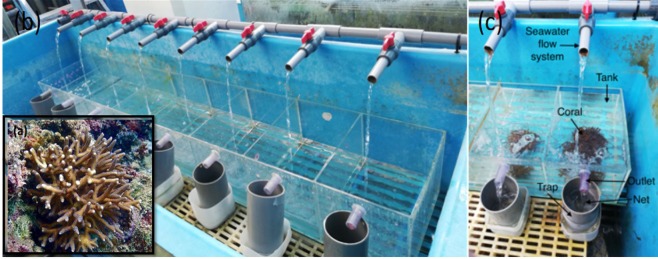
A specimen of *Seriatopora caliendrum* sampled by SCUBA divers (**a**), the coral larvae hatchery (**b**), and details of the water flow throughout the hatchery (**c**).

Coral reefs are ecologically and geologically complex marine communities that host a wealth of biodiversity and provide over US$375 billion per year in goods and services^1,2^. Unfortunately, coral reefs are suffering due to numerous anthropogenic insults that manifest across local and global scales^3,4^. Local threats, including marine pollution^5,6^, and global threats like climate change (and particularly rising seawater temperatures)^7^ have led to the need for better management aimed at conserving these fragile ecosystems^8^. Gamete cryopreservation (i.e., the use of extremely low temperatures to stabilize biologically active material indefinitely) is one means of conserving living coral tissues^9–12^, and we currently have the capacity to cryopreserve coral sperm^10,13,14^. Ideally, coral oocytes, or even larvae, could be cryopreserved, but limited success has been met with such materials, potentially due to corals’ poorly permeable membranes and high lipid content^15–17^. Oocytes of gorgonian corals, however, including *Junceella juncea*, *J. fragilis*, and *Ellisella robusta*, have been successfully cryopreserved using vitrification^18,19^, which has become increasingly popular in recent years with otherwise difficult-to-cryopreserve tissues^20^. Upon infiltrating the tissues with a high concentration of cryoprotectants (CPAs), the biological material is then supercooled at an ultra-fast cooling rate that thwarts detrimental intracellular ice formation^21,22^. To avoid recrystallization and possible damage during thawing, it is of equal importance to thaw rapidly; this can be achieved using laser warming^23^. Recent breakthroughs in vitrification and laser warming technologies have enabled the successful cryopreservation of zebrafish embryos^24^ and symbiont-free coral larvae^25^ for the first time. We hypothesized that this approach could be used to cryopreserve the larvae of the common Indo-Pacific reef coral *Seriatopora caliendrum*, which contain symbiotic dinoflagellates (family *Symbiodinaceae*) inherited from the parental colony (Fig. 1).

## Methods

### Coral larval collection

**Figure 2:**
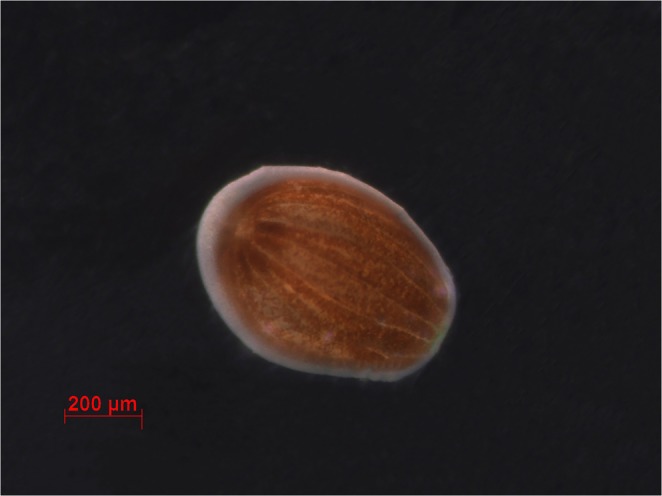
A *Seriatopora caliendrum* larva used for vitrification and laser warming.

SCUBA divers collected *S. caliendrum* (Fig. 1a) colonies at 10-m depth from the reef flats of Kenting National Park (KNP), Nanwan, Taiwan (GPS coordinates: 21°56′N, 120°44′E) upon obtaining approval from the KNP management office (permit number:10803191125) in 2018 and 2019. The corals were transported immediately to the husbandry center of the National Museum of Marine Biology and Aquarium and kept individually in 5-L tanks with continuous seawater flow (Fig. 1b) at 25 °C. On the upper part of each tanks there was an outlet that allowed seawater to exit. We placed handmade traps consisting of tubes with nets on the lower faces (Fig. 1c) beneath each outlet. The larvae were naturally spawned between 4–6 AM. After spawning, traps containing coral larvae (Fig. 2) were transported to the laboratory, and the larvae were washed thrice with filtered seawater (FSW) prepared by filtering raw seawater through a 47- mm glass fiber filter with mesh size 0.2 µm (PALL, USA) via a Rocker 300 (Taiwan) vacuum pump. Pelagic phase larvae were used within six hours of release.

(EG), (PG), dimethyl sulfoxide (DMSO), (GLY), and (METH).

### CPA toxicity

Larvae were incubated in 1 ml of 0.5, 1, 2, or 3-M concentrations of ethylene glycol (EG; JT Baker, Phillipsburg, USA), propylene glycol (PG; JT Baker), DMSO (Sigma-Aldrich, St. Louis, USA), glycerol (GLY; Darmstadt, Germany), or methanol (METH; Darmstadt) (five larvae per treatment) for 10, 20, or 30 min (n = 3 experiments) at 25 °C and the experiment was repeated three times. After CPA exposure, larvae (including controls incubated in FSW) were washed thrice with FSW, and ATP concentration was measured in one subset (with the others allowed to settle as described below). ATP concentration was measured using a cell viability assay kit (BioThema, Sweden) and a Lumat 9507 luminometer (Berthold Technologies, Germany). Larvae were mixed with 50 µL of light-stable ATP reagent for 3 min, and ATP concentration was estimated as recommended by the manufacturer.

### Gold nanobar synthesis

GNB synthesis followed a prior protocol^26^ and featured preparation of: (1) seed solution; (2) growth solution; and (3) the GNBs themselves. First, 5 mL of 0.1 mM HAuCl_4_ (Alfa Aeser; Haverhill, USA) were mixed with 5 mL of cetyltrimethylammonium bromide (0.20 M; Sigma-Aldrich). Then, 0.6 mL of 0.01 M NaBH_4_ solution (Sigma-Aldrich) were added, and the resulting solution was mixed for 20 min, at which point an orange-brown color appeared (indicative of gold nanoparticle formation; i.e., the seed solution). Next, 50 mL of 1 mM HAuCl_4_ were added to 50 mL of 0.20 M CTAB and stirred for 3 min. Then, 1 mL of 4 × 10^−3^ M was added to the solution and stirred for 10 min. Next, 2 mL of 0.0788 M ascorbic acid (Sigma-Aldrich) were added to the solution; upon stirring, the solution changed from yellow to colorless (indicating reduction of Au(III) to Au(I); i.e., the growth solution). Then, 0.3 mL of seed solution were added to the growth solution, incubated at room temperature (RT) for 2 hr, and centrifuged at 7370 × *g* for 20 min. The precipitate was re-suspended in 100 mL of Milli-Q water to obtain the GNB colloidal solution. A 2 µL drop of this solution was then loaded onto a single nickel ovular slot (1 × 2 mm) coated with formvar/carbon (Bar-Naor Ltd., Israel) and then air-dried at RT. Digital images were acquired with a Hitachi 600 transmission electron microscope (JEM-1400, JEOL, Japan) connected to a camera system (Gatan digital micrograph) at an accelerating voltage of 82 kV (filament, 56 µA).

### Vitrification and laser warming

Based on the CPA toxicity data, two vitrification solutions (VS) were designed; VS1 consisted of 2 M EG, 1 M PG, 40% (w/v) Ficoll, and 10% (v/v) of GNBs in FSW (final concentration of 1.2 × 10^18^ particles/mL and an optimized emission wavelength of 535 nm). VS2 consisted of 2 M EG, 1 M DMSO, 40% m/V Ficoll, and 10% v/v of GNBs in (same properties as for VS1) in FSW. Equilibration solutions were prepared by diluting VS solutions at ratios of 1:3 (ES1) and 1:1 (ES2) to create quarter- and half-strength VS solutions respectively. Individual coral larvae were first exposed to a 10-µL drop of ES1 for 4 min, followed by a 10-uL drop of ES2 for 2 min, and then, finally, the VS [0.8 µL; One drop is 0.8 ul, and a *S. caliedrum* larva is approximately 400 × 400 × 400 um = 0.064 µl (maximum since some shrinkage occurs in VS). Therefore, an 0.8-µl droplet can accommodate a single larval cell], in order to reduce the potential for osmotic shock or other potentially detrimental effects of the VS on the coral larvae (this exposition was conducted at 25 °C). The larva in the final drop was placed on a Kitazato Vitrification Cryotop® (Japan) and plunged into liquid nitrogen (LN2) after 1 min exposure to the VS. A custom-made device was utilized to lower the Cryotop into LN2 and to raise the sample on the aim of a single laser pulse for warming. The vitrified samples were warmed using a bench-top iWeld 980 Series, 60 joule, Nd:YAG infrared laser (LaserStar Technologies Corporation, Riverside, USA). The wavelength of the beam was 1064 nm, and the laser was equipped with a stereomicroscope to allow for the alignment of samples with the laser beam. The laser settings utilized in this study were 300 V and 10 ms pulse width with a 2-mm laser beam diameter. If the sample was vitrified during the warming stage, it was rehydrated at 25 °C in stepwise fashion by transferring the larva to 10 µl of ES2 for 2 min, followed by 10 µl of ES1 for 4 min, and finally FSW.

### Larval movement and settlement

Laser-warmed larvae were washed thrice with FSW, and larvae successfully subjected to vitrification and warming without ice crystal formation (verified by naked eye under the laser microscope) were collected individually in a 6-well Petri dish (Alpha Plus, Taiwan). Vitality (percentage of swimming and/or settled larvae over the entire larval sample size) and integrity (([number of swimming and⁄or settled larvae × 1] + [number of intact, immobile larvae × 0.5]))/(number of experimental larvae) × 100) were determined after 0.5, 1, 3, 6, 12, and 24 hr under a SteREO Discovery.V8 (Zeiss, USA) stereomicroscope. Coral larvae could swim and/or settle were scored as 1, with those intact but immobile scored as 0.5.

### Statistical analysis

All statistical analyses were undertaken with SPSS (version 17.0; Chicago, IL, USA), and one-sample Kolmogorov-Smirnov tests were used to ensure that the data were normally distributed. One-way ANOVAs were then performed upon confirming homogeneity of variance with Levene’s tests (*p* > 0.05). When there were differences detected by ANOVA, Tukey’s *post-hoc* tests were used to identify individual mean differences. All data were presented as mean ± SEM across the three replicates and *p* values < 0.05 were considered to be significant.

## Results

**Figure 3:**
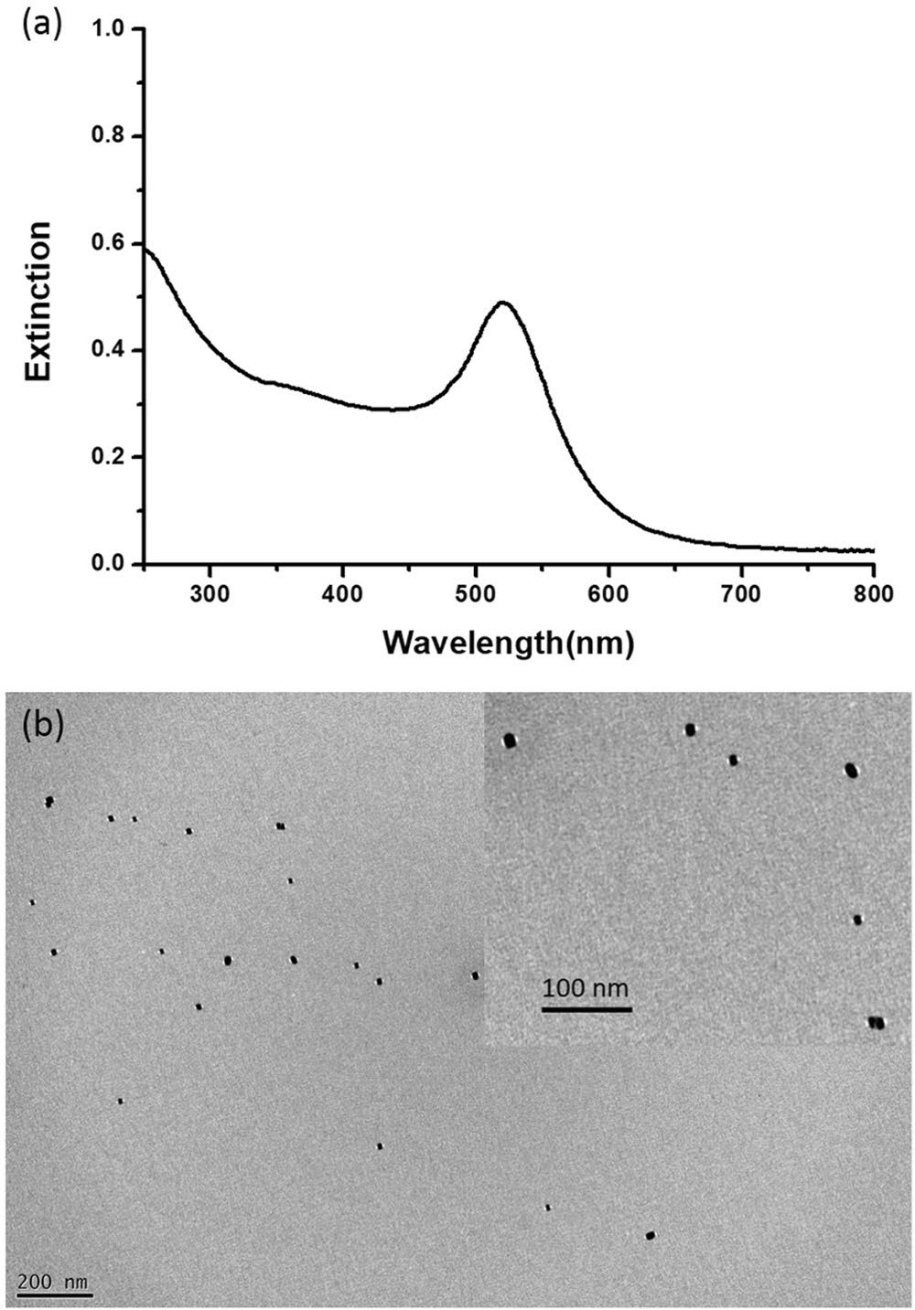
Gold nanobars (GNBs) used for laser warming. (**a**) The UV-vis-NIR spectrum of the GNB colloids; (**b**) Transmission electron microscopy (TEM) image of GNBs. Figure of High magnification (100 K) shows in the top-right corner.

The UV-vis-NIR spectrum of the GNB colloids revealed a peak at ~535 nm (Fig. 3a), presumably corresponding to the longitudinal dipolar localized surface plasmon resonance (LSPR) mode of the GNBs. The peak position, which is quite close to the LSPR band of the spherical gold nanoparticles (13-nm in diameter), indicates that the aspect ratio of the GNBs prepared was close to 1, consistent with the TEM image (Fig. 3b).

**Figure 4:**
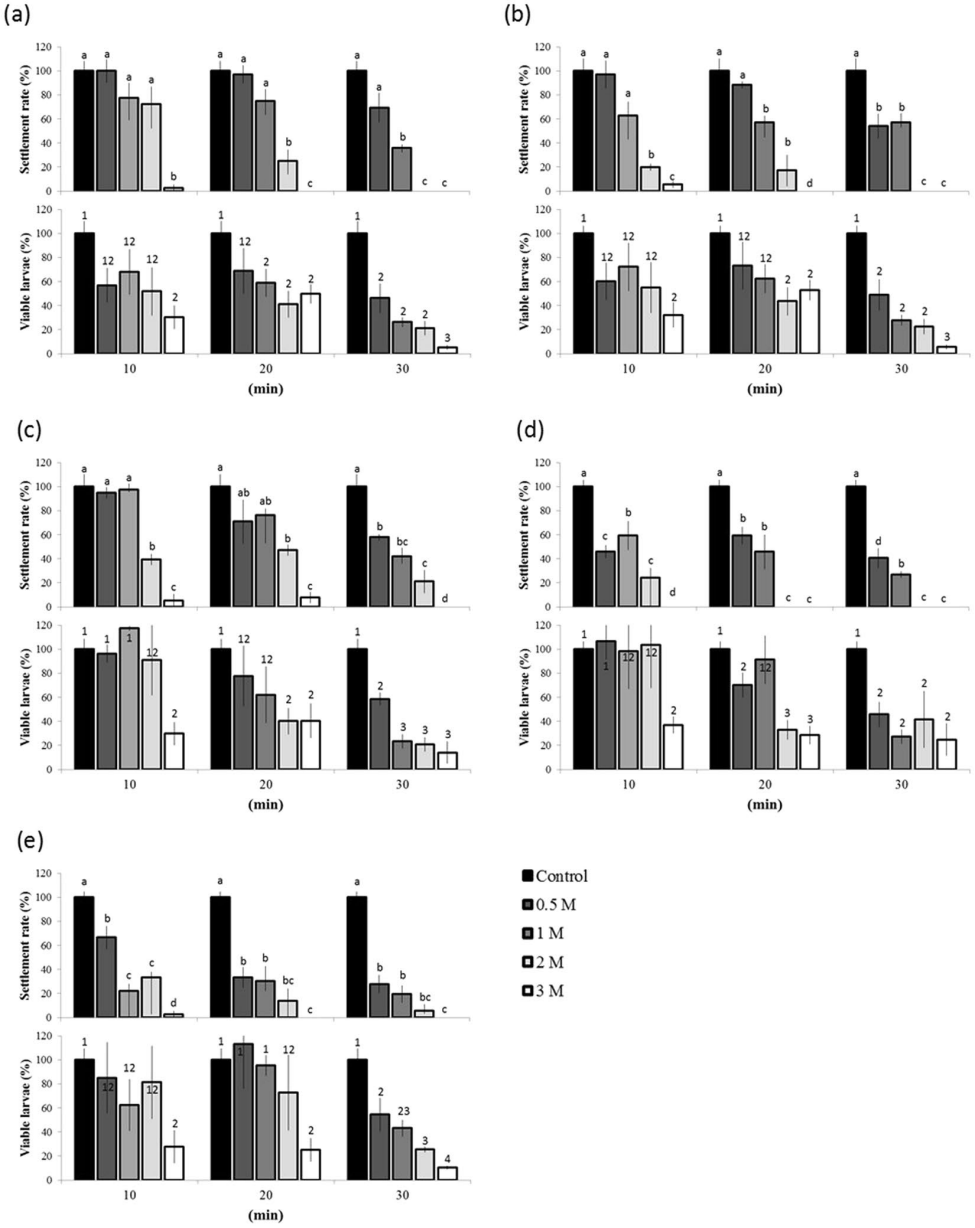
Toxic effects (mean +/− SEM) of different cryoprotectants (CPA) on larval viability (top row of each panel) and settlement rate (bottom row of each panel) of *Seriatopora caliendrum* larvae: ethylene glycol (EG; **a**), propylene glycol (PG; 2), DMSO (**c**), glycerol (GLY; **d**), and methanol (METH; **e**). Each CPA was used at concentrations of 0.5, 1, 2, or 3 M for 10, 20, or 30 min. The bars with different numbers and letters on top, respectively, indicate significant differences (Tukey’s *p* < 0.05) in ATP content and settlement rate, respectively. Viability was referred by ATP content.

Cryoprotectant toxicity towards *S. caliendrum* larvae generally increased with increasing concentrations and exposure times (Fig. 4), as gauged by larval viability and settlement. ATP is critical to show that larvae maintain the ability to generate ATP upon CPA treatment. Therefore, ATP content was referred to viability. The least toxic CPAs were EG (Fig. 4a; 0.5–2 M), PG (Fig. 4b; 0.5–1 M), and DMSO (Fig. 4c; 0.5–1 M), none of which led to significant changes in these response variables relative to controls after 10 min of exposure. The use of 1 M METH (Fig. 4e; most toxic) and GLY (Fig. 4d; next-to-most toxic), on the other hand, led to significantly lower settlement rates (*p* < 0.05) after 10 min. The highest settlement rate was achieved upon using 1 M EG: 68 and 59% after 10 and 20 min (Fig. 4a; respectively. Low settlement rates were observed in larvae incubated in 3 M CPA. Regarding the ATP content, 10-min exposures to 0.5, 1, and 2 M of DMSO (Fig. 4c), GLY (Fig. 4d), and METH (Fig. 4e) induced increases in ATP content relative to controls (though the results were not statistically significant; *p* > 0.05). The same trend occurred after 20 min, except for 1 M, 2 M, and 3 M concentrations of DMSO, GLY, and all CPAs, respectively, after 30 min of exposure (which led to lower ATP content).

**Figure 5:**
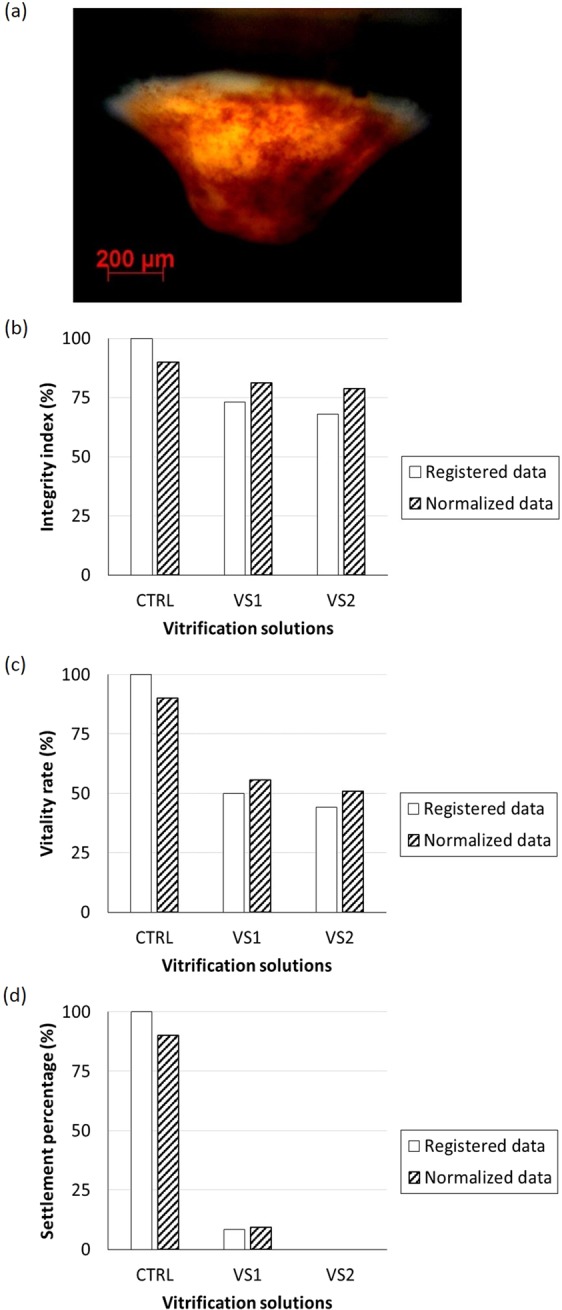
A *Seriatopora caliendrum* larva (**a**) that settled 12 hours after vitrification and warming using vitrification solution 1 (VS1). Integrity of vitrified and laser-warmed larvae (**b**; as an index; see methods), vitality (**c**; as a rate), and settlement (**d**; %) represented as registered (raw) and normalized data.

The concentration and type of cryoprotectant selected for VS was based on the results of the toxicity study; CPAs that did not elicit a significantly different effect were chosen. Vitrification and laser warming utilizing vitrification solutions VS1 and VS2 resulted in 50% (24/48) and 44% (22/50) of larvae resuming swimming, respectively (Table 1). Of the larvae that resumed swimming, 4 larvae that were vitrified and laser warmed using VS1 settled (8.5% of the initial 48; Fig. 5a,b). Although VS2 scored slightly lower than VS1 in terms of larval integrity (Fig. 5b; 81 and 78% for VS1 and VS2, respectively) and vitality (Fig. 5c; 55 and 50%, respectively) after vitrification and warming, none of the VS2 larvae that were vitrified and laser-warmed settled (Fig. 5d); VS1 was, therefore, superior.

**Table 1:** The effects of two vitrification solutions (VS) on coral larval motion, settlement, and morphology after vitrification and laser warming.

VS used	Total larvae used	Mobile larvae	Settled larvae	Intact, mobile larvae	Damaged larvae
VS1	48 (100%)	24 (50%)	4 (8%)	22 (46%)	2 (4%)
VS2	50 (100%)	22 (44%)	0	24 (48%)	4 (8%)

## Discussion

This study represents the first successful attempt to cryopreserve coral larvae containing the dinoflagellate endosymbionts that are vital to their survival. It is also important to note that the larvae used in the present study were around twice the size (~450 µm) as coral larvae that have previously been successfully cryopreserved (*Fungia scutaria*, ~200 µm^25^). Most *Acropora* species (the main reef-building coral on coral reefs around the world) have larvae that are around 500 µm or larger in size, so the ability to cryopreserve coral larvae in this size range will be crucial to the ability to preserve the genetics of these species in biorepositories. The coral species used in the present study may therefore be a useful model species for the continued development of laser warming technology for application in threatened coral species worldwide.

We attribute the success achieved in the present study, to some extent, to the use of multiple CPAs, which can act to dampen the toxic effects of either in isolation^27^. Specifically, EG, PG, and DMSO were mixed into one VS; all three having been used successfully in cryopreservation efforts of other invertebrate material^28–30^ (including gorgonian coral oocytes^18^ and coral larvae^25^). Upon combining EG and PG, a sufficiently viscous VS was generated, which may have permitted the successful vitrification that ultimately led to larvae that were competent upon thawing. During vitrification, it is important to limit the degree of osmotic stress to which samples are exposed during VS equilibration and removal, as such stress can damage cell membranes and lead to cell death^31^. One means of mitigating this problem is the stepwise addition and removal of VS through the use of equilibration solutions (ES)^20^. The VS composition, exposure time and equilibration strategy adopted in this study generated an osmotic pressure that allowed an adequate removal of intracellular water content. This dehydration enhanced the ability of the coral larva to survive vitrification and subsequent warming by minimizing intracellular ice crystal formation^23^. Equilibration strategies have successfully prevented osmotic shock by gradual dehydration in prior works^18,32^ and may have contributed to the success of the method developed herein.

Droplet volume is another factor that can affect the success of vitrification both during cooling and warming phases; the smaller the droplets of CPAs with sample, the less likely it is that ice crystal will form^32,33^. Therefore, vitrification drops of only 0.8 uL were used. During warming, the energy of the laser that hits the droplet must be uniform enough to avoid large thermal gradients; otherwise, membrane damages may occur in the sample^34^. GNBs were used to homogenize the heat distribution of the laser on the droplet, and it was found that the optimal GNB concentration was 1.2 × 10^18^ particles/mL (data not shown) in combination with a voltage of 300 V, pulse width of 10 ms, and a laser beam diameter of 2.0 mm. However, light effects on GNBs must also be considered, and GNBs must have a resonance energy in line with the wavelength of the laser beam in order to generate a homogeneous heat dissipation^25^. The interaction between light and GNBs is given by the surface plasmon resonance^35^, and prior works have revealed that the maximum optical torque between the laser and GNB wavelengths can cause severe deformation, and even melting, of the latter^36^. Therefore, a laser with an off-resonant and red-shifted wavelength (1064 nm) was used with GNBs (535 nm); this yielded a sufficient plasmonic resonance for uniformly warming the sample in a way in which GNBs were not damaged.

VS composition and exposure, droplet volume, laser beam voltage and diameter, and GNB resonance energy were all considered in protocol development, though, despite having optimized all such parameters, we surmise that an improved, less invasive protocol could be devised. This stems from our observation that, although a small percentage of cryopreserved larvae could settle and maintain brown coloration for a week thereafter (despite a gradual loss of endosymbionts), they ultimately died. Control larvae were also placed in a petri dish that was then immersed in an aquarium in the husbandry facility, and they survived approximately one month. Perhaps lingering heat damage from the fast-thawing process led to macromolecular damage to the larvae or algal symbionts that could not readily be repaired by the larval recruits. The higher ATP content in the CPA-inoculated larvae could also attest to a prolonged cellular response to the cryoprotectant process, as has been documented in coral oocytes and coral endosymbiont *Symbiodinum*^37–40^. Regardless of the explanation, this first study of cryopreserved endosymbiont-bearing larvae constitutes one mechanism by which coral biological material may be conserved over prolonged time periods, and this vitrification approach may prove successful with larvae from other reef-building corals to where “cryo-arks” could be developed for storage of biological material to be later used to reseed marine ecosystems.
